# Acute and long-term effects of cannabinoids on hypertension and kidney injury

**DOI:** 10.1038/s41598-022-09902-6

**Published:** 2022-04-12

**Authors:** Daria Golosova, Vladislav Levchenko, Olha Kravtsova, Oleg Palygin, Alexander Staruschenko

**Affiliations:** 1 Department of Physiology, Medical College of Wisconsin, 8701 Watertown Plank Road, Milwaukee, WI 53226 USA; 2 Department of Molecular Pharmacology and Physiology, University of South Florida, 560 Channelside Dr., Tampa, FL 33602 USA; 3 Division of Nephrology, Department of Medicine, Medical University of South Carolina, Charleston, SC 29425 USA; 4 Hypertension and Kidney Research Center, University of South Florida, Tampa, FL 33602 USA; 5 Clement J. Zablocki VA Medical Center, Milwaukee, WI 53295 USA

**Keywords:** Physiology, Kidney

## Abstract

Cannabinoids and their endogenous and synthetic analogs impact blood pressure and contribute to the incidence of hypertension. It was previously reported that the endocannabinoid system plays an important role in developing hypertension; however, it was also shown that cannabinoids elicit profound hypotension associated with hemorrhagic, cardiogenic, and endotoxic shock. This study aimed to test acute and chronic effects of an endogenous ligand of cannabinoid receptor anandamide (AEA) on blood pressure and kidney injury in vivo in conscious Dahl salt-sensitive (SS) rats. We demonstrated that acute *i.v.* bolus administration of a low or a high doses (0.05 or 3 mg/kg) of AEA did not affect blood pressure for 2 h after the injection in Dahl SS rats fed a normal salt diet (0.4% NaCl). Neither low nor high doses of AEA had any beneficial effects on blood pressure or kidney function. Furthermore, hypertensive rats fed a HS diet (8% NaCl) and chronically treated with 3 mg/kg of AEA exhibited a significant increase in blood pressure accompanied by increased renal interstitial fibrosis and glomerular damage at the late stage of hypertension. Western blot analyses revealed increased expression of Smad3 protein levels in the kidney cortex in response to chronic treatment with a high AEA dose. Therefore, TGF-β1/Smad3 signaling pathway may play a crucial role in kidney injury in SS hypertension during chronic treatment with AEA. Collectively, these data indicate that prolonged stimulation of cannabinoid receptors may result in aggravation of hypertension and kidney damage.

## Introduction

The endogenous cannabinoids, together with cannabinoid receptors CB1 and CB2 and the enzymes involved in their metabolism, form the endocannabinoid system (ECS). Multiple studies in the last two decades have led to the recognition that both CB1 and CB2 receptors are present not only in the central nervous system but in many other peripheral organs where they play a vital role in a vast array of pathophysiological processes, including oxidative stress, inflammation, and fibrogenesis^1^.

The natural ligands of CB1 and CB2 receptors, arachidonoyl ethanolamide or anandamide (AEA) and 2-arachidonoylglycerol (2-AG)^2^, are lipid-like substances called endocannabinoids. The primary active constituent of cannabis drugs is Δ^9^-Tetrahydrocannabinol (THC) that exerts its function through binding to two cannabinoid receptors, CB1 and CB2. THC mimics the actions of the endocannabinoids AEA and 2-AG^3,4^. Several studies showed that CB1 and CB2 receptors are expressed in rodent and human kidneys^5–13^ revealing a full ECS^6,14–16^. ECS is an essential player in the pathogenesis of diabetic nephropathy, drug nephrotoxicity, and progressive chronic kidney disease (CKD)^17–19^. CB1 and CB2 receptors are G protein-coupled receptors (GPCRs), and their activation relies on the G protein-mediated receptor phosphorylation and recruitment of β-arrestins, the scaffold proteins for receptor desensitization, internalization, and trafficking^20,21^. β-arrestin 1 is responsible for GPCR activation and downstream signal transduction via Src, AKT, extracellular signal-regulated protein kinase (ERK1/2), and other kinases^20^. As a G_i/o_-coupled GPCR, the activation of CB receptors leads to the inhibition of adenylate cyclases (AC) via G_i_ subunits^22^. AEA was also shown to be a substrate for a cytochrome P450 mediated processes^23^. Cytochromes P450 oxidize arachidonic acid (AA) to the physiologically active molecules hydroxyeicosatetraenoic acids (HETEs) and epoxyeicosatrienoic acids (EETs) playing important roles in blood pressure regulation and inflammation^24–29^.

Cannabinoids and their endogenous and synthetic analogs were implicated in the regulation of blood pressure. Previous studies revealed that short-term administration of endocannabinoids had some beneficial effects on blood pressure^30–32^. A number of studies describe that cannabinoids and their endogenous and synthetic analogs exert hypotensive and cardiodepressor effects by complex mechanisms involving direct and indirect effects on myocardium and vasculature^33^. In studies in conscious humans, an acute dose of THC elicited dose-related tachycardia without altering blood pressure^34^. Chronic use of cannabinoids in normotensive men elicit a long-lasting decrease in blood pressure and heart rate^35^. In anesthetized rats acute administration of cannabinoids, including AEA, produced hypotension and bradycardia and a dose-dependent drop in arterial pressure^30,36,37^. A separate report showed that in normotensive conscious rats AEA induced a transient increase in the mean arterial blood pressure^38^. Therefore, it became apparent that complex cardiovascular actions of AEA are not consistent and need to be further investigated.

Herein, we used Dahl salt-sensitive (SS) rats to investigate blood pressure changes and kidney function in response to acute and chronic treatment with AEA. The objective of the present study was to perform a detailed characterization of the effects of AEA in Dahl SS rats on a normal salt diet or a high salt diet during the development of salt-induced hypertension. We demonstrate that 1) acute injection of AEA did not affect blood pressure in conscious Dahl SS rats; 2) chronic treatment with a low dose of AEA (0.05 mg/kg) did not modulate blood pressure or kidney function during the development of SS hypertension; 3) a high dose of AEA (3 mg/kg daily) led to a significant aggravation of hypertension and profound renal damage after chronic administration; 4) the negative effect of chronic treatment with AEA was correlated with the upregulated Smad3 intracellular pathway. Collectively, these data provide novel insight into the chronic effects of AEA on developing salt-induced hypertension and kidney damage.

## Results

### Acute injection of anandamide does not change mean arterial pressure in Dahl SS rats on a normal salt diet

**Figure 1:**
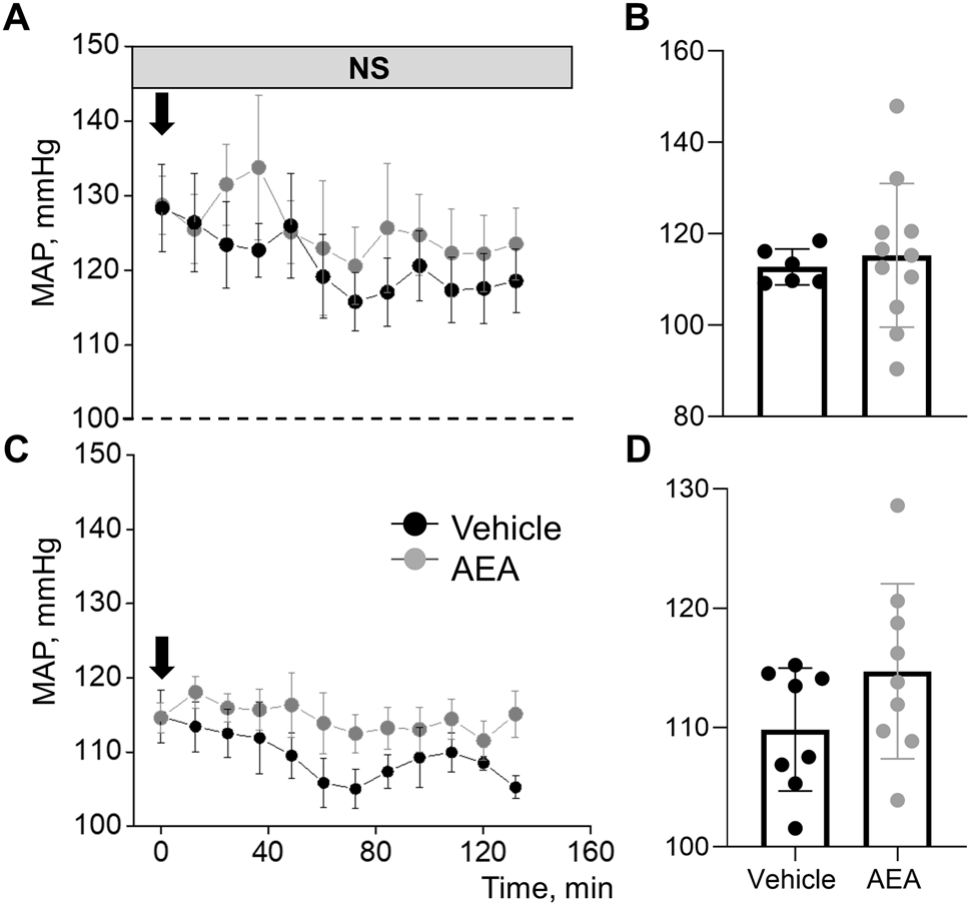
Acute treatment with anandamide (AEA) does not affect blood pressure in Dahl SS rats on a normal salt diet. Mean arterial pressure (MAP) in male SS rats after a single injection of AEA at a dose 0.05 mg/kg (**A, B**) and 3 mg/kg (**C, D**) or a corresponding vehicle in SS rats on a normal salt (NS, 0.4% NaCl) diet. (**B, D**) Summary graphs of average MAP 2 h after AEA administration in a low (**B**) or a high (**D**) doses.

To test the acute effects of AEA we used a single *i.v*. bolus injection of the drug to Dahl SS rats fed a normal salt diet (NS). The drug was used at a low (Fig. 1A,B) or a high doses (Fig. 1C,D) according to previously reported studies^37–39^. Mean arterial pressure (MAP) was monitored through the intraarterial catheter connected to a pressure transducer and averaged every 15 min for two hours following the drug injections. We did not observe acute effects of AEA on MAP at either a low or high doses (Fig. 1).

### Evaluation of the effect of chronic treatment with Anandamide on the development of blood pressure and kidney injury in Dahl SS rats on a high salt diet

**Figure 2:**
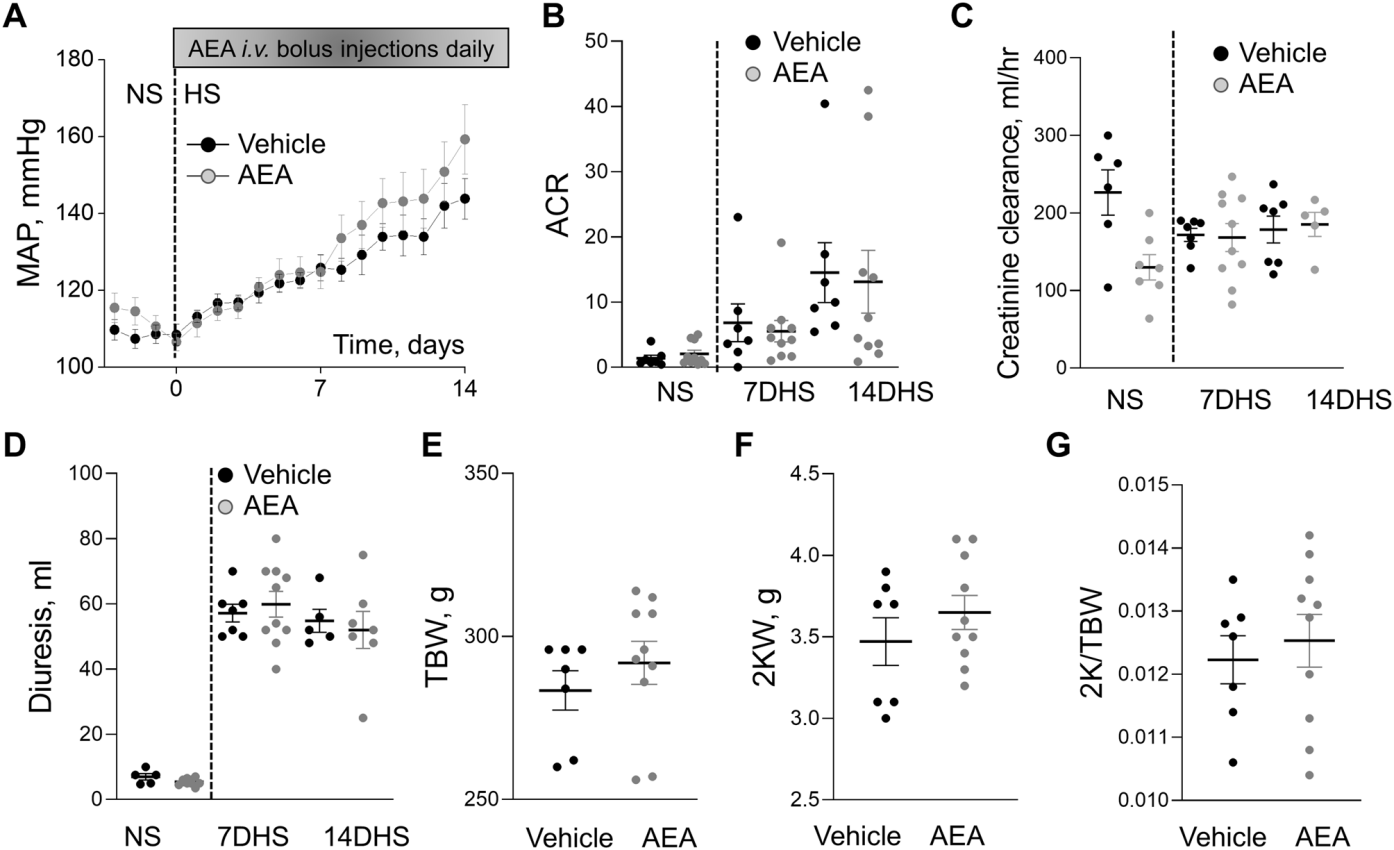
Effect of chronic treatment with AEA at a dose 0.05 mg/kg on the development of salt-induced hypertension in Dahl SS rats. (**A**) Changes in the development of mean arterial pressure (MAP) in male SS rats following a change in a diet from a normal salt (NS, 0.4%; day 0) to a HS (8% NaCl) and chronically treated with AEA at a dose 0.05 mg/kg (n = 9) or vehicle (n = 7) (ANOVA, p < 0.05). (**B**) Albumin/creatinine ratio (ACR, Alb/Cre, 24 h collection). (**C**) Creatinine clearance (creatinine urine concentration x urine flow / creatinine serum concentration). (**D-G**) Diuresis (**D**), total body weight (TBW, **E**), 2 kidneys weight (2KW, **F**), and 2 kidneys to body weight ratio (2 K/TBW, **G**) changes in these groups. For the data sets, urine samples were collected for 24 h. n ≥ 5 rats per group.

**Figure 3:**
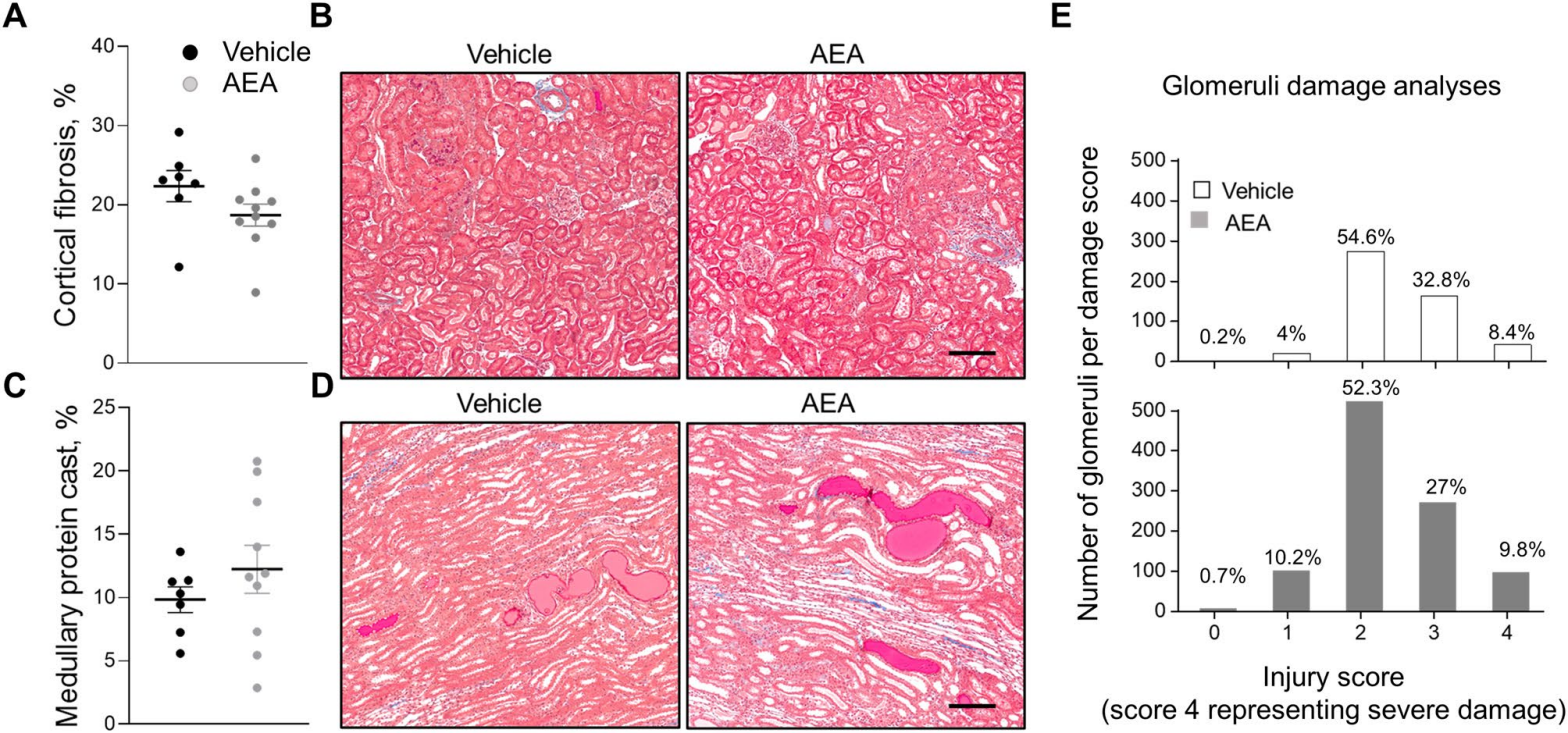
Development of the kidney injury after chronic exposure to a low dose of AEA (0.05 mg/kg) or vehicle in Dahl SS rats fed a high salt diet. (**A, C**) Summary graphs of cortical fibrosis (**A**) and the medullary protein cast area (percent total kidney area) (**C**). Protein cast analysis was performed using a color thresholding method using Metamorph software. Fibrosis was assessed using color deconvolution in the Fiji image application (ImageJ 1.47v, NIH). (**B, D**) Representative images of kidney tissue stained with Masson’s trichrome (× 10 magnification). Scale bar is 150 µm. (**E**) Glomerular injury score (0–4, where 0 = no damage) assessed by semiquantitative morphometric analysis. Numbers of glomeruli per score are shown on the y-axes. The percentage of glomeruli within the selected score range defined from cumulative distribution is shown above the corresponding column. n ≥ 5 rats per group, N ≥ 500 glomeruli per group. Statistical analysis consisted of a Kolmogorov–Smirnov test to identify significant differences between the groups (OriginPro 9.0) with a *P* > 0.05, distributions were not significantly different.

To determine the effects of chronic exposure to AEA on blood pressure and renal damage during the development of SS hypertension, we used Dahl SS rats fed a high salt (HS) diet. The experimental protocol is shown in Supplementary Figure S1A. The dietary sodium content was changed from NS to HS, which caused profound elevation of blood pressure in rats accompanied by the rapid development of renal damage. To test the effect of chronic AEA exposure, animals were administered 0.05 mg/kg *i.v.* bolus injections of AEA or vehicle daily from the start of a HS protocol. Chronic treatment with a low dose of AEA exhibited a consistent trend to increase MAP at the second week of the HS protocol (Fig. 2A). We found no significant differences in the development of albuminuria (Fig. 2B) or creatinine clearance (Fig. 2C) between vehicle-treated and AEA-treated groups. Other renal function parameters like diuresis (Fig. 2D), urine and blood electrolytes, and glucose (Tables 1 and 2) were not different between the groups. The treatment did not affect total body weight (TBW, Fig. 2E), kidney weight (2KW, Fig. 2F), and kidney to body weight ratio (2 K/TBW, Fig. 2G). During the HS challenge in Dahl SS rats, the renal lesion was characterized by hyaline (protein) casts and cortical fibrosis, which were highly present in both groups. Figures 3A,C show a summarized analysis of kidney interstitial fibrosis and medullary casts. Figures 3B,D display representative images of medullary and cortical areas of both groups. Qualitative and statistical comparisons of glomerular injury between the two groups suggesting that the small dose of AEA did not promote any significant changes in the glomeruli injury score are shown in Fig. 3E.

**Table 1:** Fractional excretion of electrolytes in SS rats during chronic experiment with or without treatment with the 0.05 mg/kg of AEA within 14 days on HS.

Fractional excretion of electrolytes, %	7DHS + Vehicle	7DHS + AEA (3 mg/kg)
Potassium	8 ± 2	10 ± 3
Sodium	4 ± 1	5 ± 1
Calcium	1.7 ± 1.1	1.5 ± 0.9
Chloride	6 ± 1	7 ± 2

14DHS	14DHS + Vehicle	14DHS + AEA (3 mg/kg)
Potassium	9 ± 2	8 ± 2
Sodium	4 ± 2	3 ± 1
Calcium	1.5 ± 0.8	1.2 ± 0.5
Chloride	6 ± 2	3.8 ± 0.5

7DHS, 14DHS – 7 days and 14 days on HS (8% NaCl). Data are presented as means ± SE; n ≥ 5 rats for each group; t-test *P < 0.05.

**Table 2:** Blood electrolytes and glucose analysis of SS rats during chronic experiment with or without treatment with the 0.05 mg/kg of AEA within 14 days on HS.

Blood electrolytes and glucose	7DHS + Vehicle	7DHS + AEA (0.05 mg/kg)
Potassium, mM	3.3 ± 0.1	3.3 ± 0.1
Sodium, mM	141 ± 1	143 ± 1
Calcium, mM	1.33 ± 0.02	1.28 ± 0.02
Chloride, mM	102 ± 1	105 ± 1
Glucose, mg/dL	126 ± 3	127 ± 2

14DHS	14DHS + Vehicle	14DHS + AEA (0.05 mg/kg)
Potassium, mM	3.3 ± 0.1	3.1 ± 0.1
Sodium, mM	142 ± 0.4	141 ± 01
Calcium, mM	1.30 ± 0.02	1.25 ± 0.02
Chloride, mM	103 ± 1	104 ± 1
Glucose, mg/dL	128 ± 23	129 ± 3

7DHS, 14DHS – 7 days and 14 days on HS (8% NaCl). Data are presented as means ± SE. n ≥ 7 rats for each group; t-test *P < 0.05.

**Figure 4:**
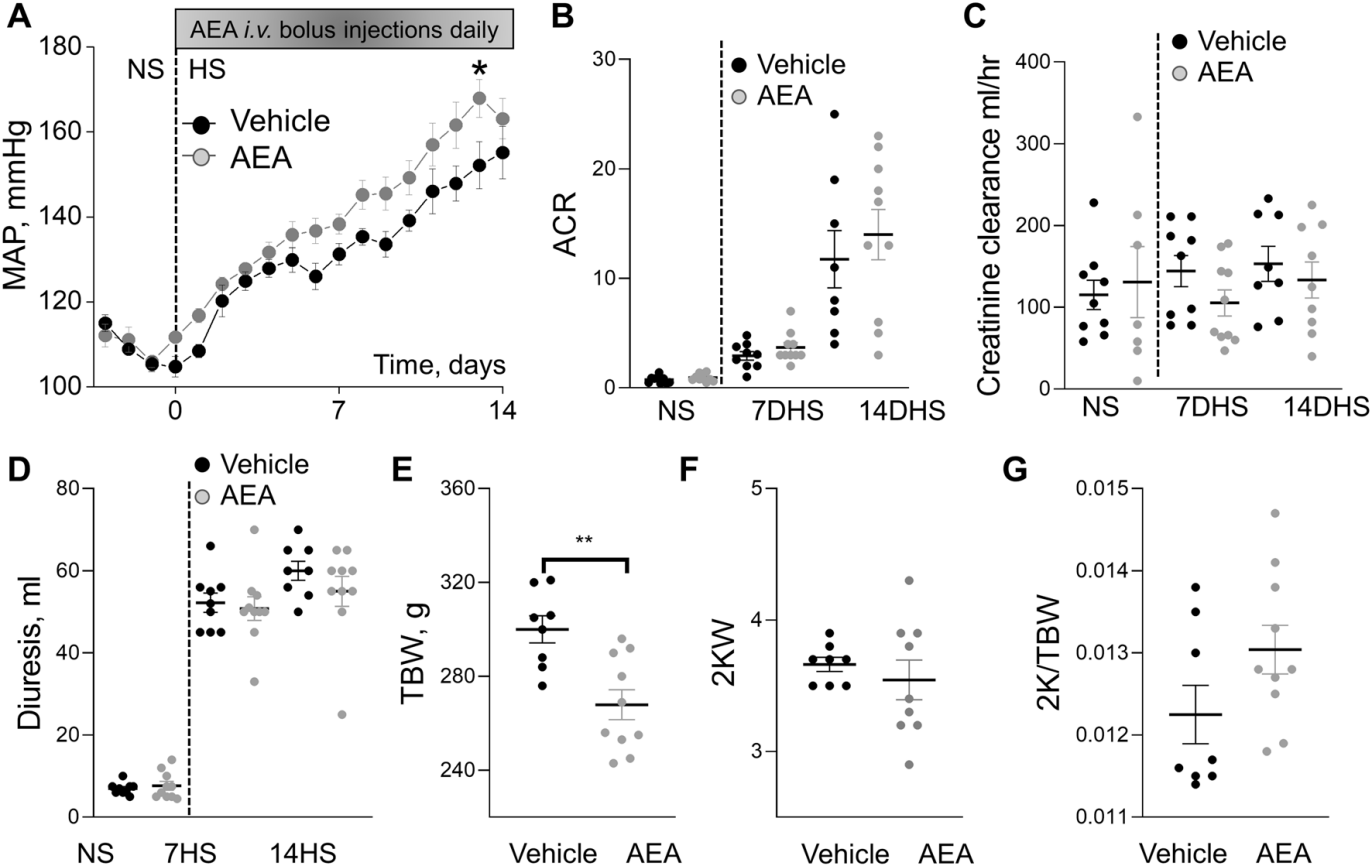
Effect of chronic treatment with a high dose of AEA (3 mg/kg) on the development of salt-induced hypertension in Dahl SS rats. (**A**) Changes in the mean arterial pressure (MAP) in SS rats following a change in a diet from a normal salt (NS, 0.4%; day 0) to a high salt (HS, 8% NaCl) and chronically treated with AEA at a dose 3 mg/kg (n = 10) or vehicle (n = 9) for 14 days. *P < 0.05. (**B**) Albumin/creatinine ratio (ACR, Alb/Cre, 24 h collection). (**C**) Creatinine clearance (creatinine urine concentration x urine flow / creatinine serum concentration). Diuresis (**D**), total body weight (TBW, **E**), 2 kidneys weight (2KW, **F**), and 2 kidneys to body weight ratio (2 K/TBW, **G**) changes in these groups. For the data sets, urine samples were collected for 24 h. n ≥ 7 rats per group. Data were analyzed using one-way ANOVA followed by Holm-Sidak post hoc test.

In the following experiments, we used a high dose of AEA. The details of the protocol are demonstrated in Supplementary Fig. S1. Blood and urinary electrolyte analyses were summarized in Tables 3 and 4. Significant changes in urinary fractional excretion of calcium during chronic cannabinoid exposure by day 7 of HS challenge was detected. However, when we evaluated urinary ion excretion per day, we did not see differences (Supplementary Tables S1-2). Plasma creatinine remained unchanged between these groups (Supplementary Fig. S1B, C). In contrast to experiments with a low dose, *i.v.* administration of a high dose of AEA induced a significant elevation in MAP at the late stage of hypertension (Fig. 4A). Albuminuria, creatinine clearance, and diuresis remained at the same level between the vehicle and AEA-treated groups (Fig. 4B–D). Both groups had no difference in body and kidney weights (Fig. 4E–G).

**Table 3:** Fractional excretion of ions during chronic treatment with 3 mg/kg of AEA within 14 days on HS.

Fractional excretion of electrolytes, %	7DHS + Vehicle	7DHS + AEA (3 mg/kg)
Potassium	12 ± 1	15 ± 2
Sodium	5 ± 1	6 ± 1
Calcium	1.6 ± 0.2	2.6 ± 0.4*
Chloride	6 ± 1	9 ± 1

14DHS	14DHS + Vehicle	14DHS + AEA (3 mg/kg)
Potassium	12 ± 1	13 ± 3
Sodium	5 ± 1	5 ± 1
Calcium	1.8 ± 0.3	1.9 ± 0.4
Chloride	7 ± 1	6 ± 1

7DHS, 14DHS – 7 days and 14 days on HS (8% NaCl). Data are presented as means ± SE. n ≥ 8 rat per group; t-test *P < 0.05.

**Table 4:** Blood electrolytes and glucose analysis of SS rats during chronic experiment with or without treatment with the 3 mg/kg of AEA within 14 days on HS.

Blood electrolytes and glucose	7DHS + Vehicle	7DHS + AEA (3 mg/kg)
Potassium, mM	3.0 ± 0.1	3.2 ± 0.1
Sodium, mM	144 ± 1	145 ± 1
Calcium, mM	1.33 ± 0.01	1.31 ± 0.01
Chloride, mM	102 ± 1	105 ± 1
Glucose, mg/dL	123 ± 2	118 ± 3

14DHS	14DHS + Vehicle	14DHS + AEA (3 mg/kg)
Potassium, mM	3.1 ± 0.01	3.1 ± 0.04
Sodium, mM	143 ± 1	143 ± 0.4
Calcium, mM	1.26 ± 0.04	1.26 ± 0.01
Chloride, mM	106 ± 3	104 ± 2
Glucose, mg/dL	121 ± 2	122 ± 3

7DHS, 14DHS – 7 days and 14 days on HS (8% NaCl). Data are presented as means ± SE. n ≥ 8 rat per group; t-test *P < 0.05.

**Figure 5:**
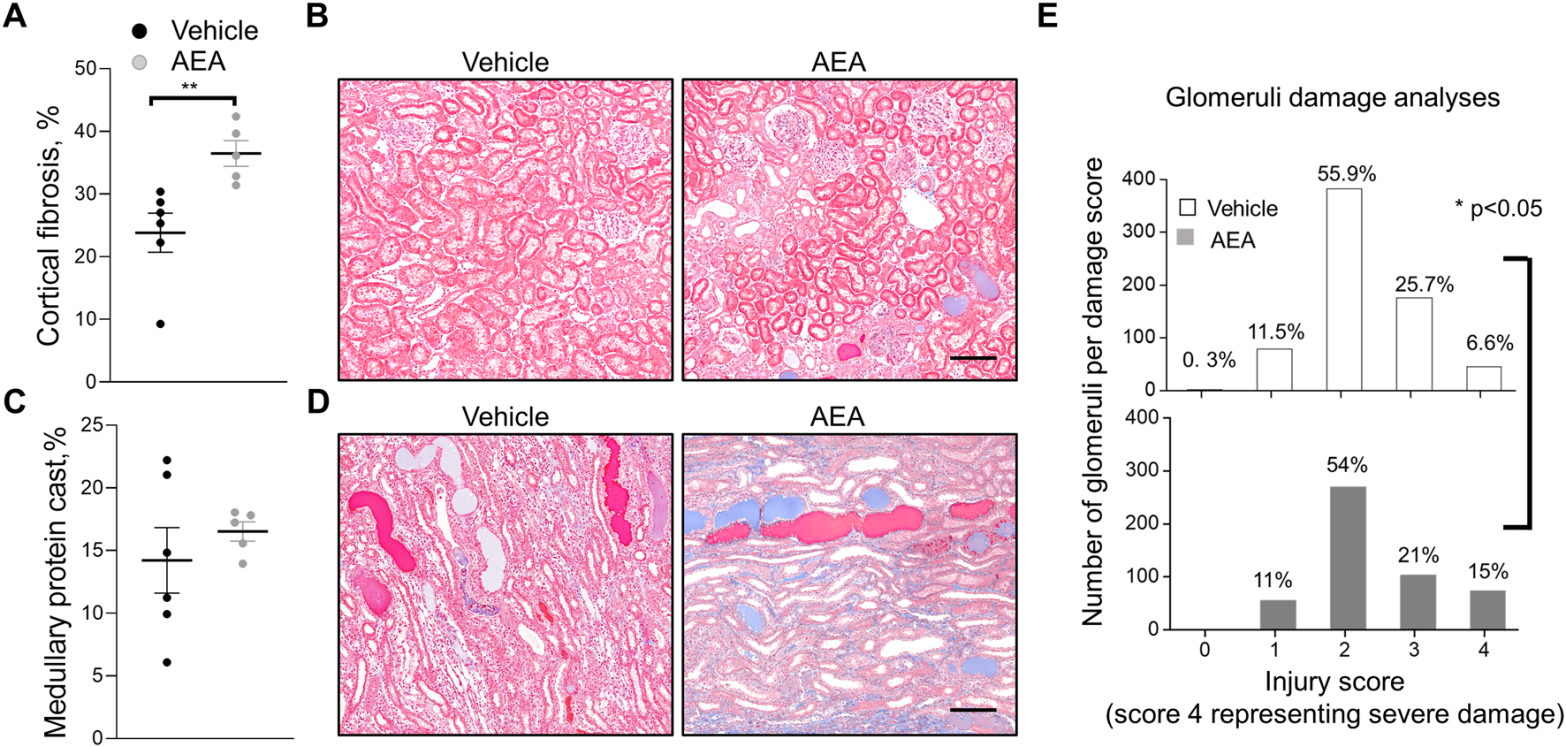
Development of the kidney injury after chronic exposure to a high dose of AEA (3 mg/kg) in Dahl SS rats. (**A, C**) Summary graphs of the cortical fibrosis (**A**) and medullary protein cast area (percent total kidney area; **C**). Protein cast analysis was performed using a color thresholding method using Metamorph software. Fibrosis was assessed using color deconvolution in the Fiji image application (ImageJ 1.47v, NIH). Data were analyzed using one-way ANOVA followed by Holm-Sidak post hoc test. (**B, D**) Representative images of cortical and medullary kidney tissue stained with Masson’s trichrome (× 10 magnification). Scale bar is 150 µm. *P < 0.05. (**E**) Glomerular injury score assessed by semiquantitative morphometric analysis. Numbers of glomeruli per score are shown on the y-axes. The percentage of glomeruli within the selected score range defined from cumulative distribution is shown above the corresponding column. Note the difference between the groups within the damage score 4. n ≥ 5 rats per group, N ≥ 500 glomeruli per group. A Kolmogorov–Smirnov test was used to identify significant differences between the groups (OriginPro 9.0) with a P < 0.05, distributions were significantly different.

During the development of salt-induced hypertension, a high dose of AEA promoted additional renal damage, reflected by an increase in interstitial fibrosis and worsened glomeruli damage score (Fig. 5A,E). Representative images with glomeruli damage scores are shown in Supplementary Figure S2. Significant changes in cortical nephrin expression were not detected between the groups (Supplementary Fig. S3). Protein cast was present in both hypertensive groups (Fig. 5C) but did not reach a statistical difference between vehicle and treated animals (Fig. 5E). Figures 5B,D show representative images of medullary and cortical areas.

### Chronic treatment with a high dose of AEA produced substantial activation of pro-fibrotic Smad3 pathway

**Figure 6:**
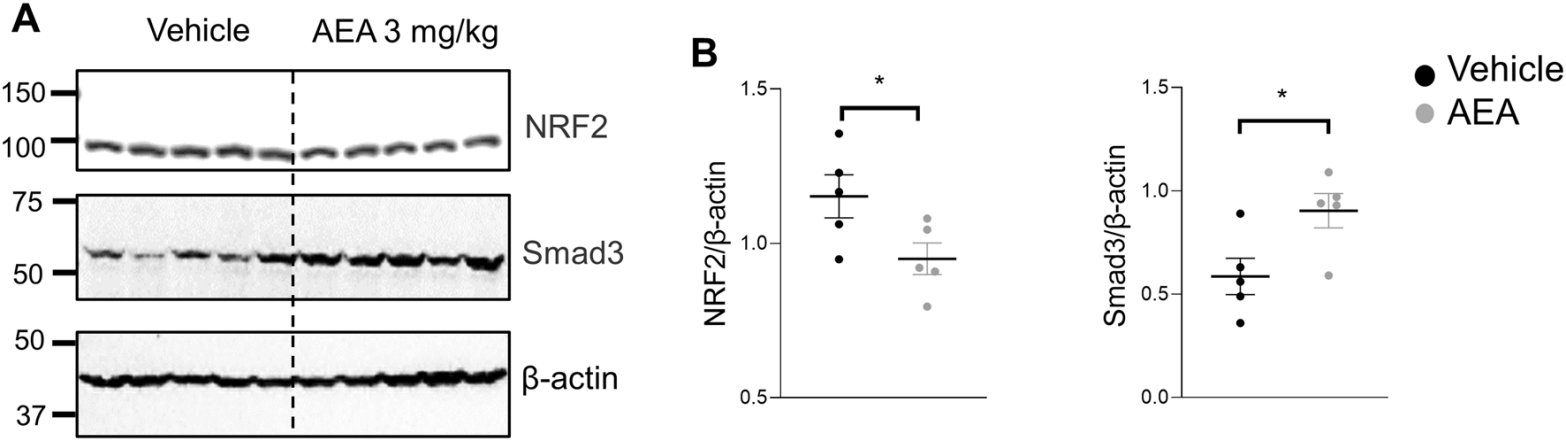
Chronic treatment with AEA at a dose 3 mg/kg leads to increased Nrf2 and Smad3 protein expression in the kidney. (**A**) Western blots showing renal expression of Nrf2, and Smad3 in the kidney cortex after chronic treatment with a high dose of AEA. (**B**) Quantitative data showing renal expression of Nrf2 and Smad3 normalized to β-actin. Data were analyzed using unpaired t-test with Welch’s correction. Full-length blots are presented in Supplementary Figure S6.

To evaluate downstream regulators of AEA-mediated intracellular pathway, we tested β-arrestin 1 expression. Chronic treatment with either dose of AEA revealed no changes neither in protein levels of β-arrestin 1 (Supplementary Fig. S3) nor its phosphorylated form (Supplementary Fig. S4). We also evaluated effects of AEA on extracellular signal-regulated kinase (ERK1/2), c-Jun N-terminal kinase (JNK) and their phosphorylated isoforms. However, neither ERK1/2 nor JNK1/2/3 protein levels changed after AEA treatment (Supplementary Fig. S4). A high AEA dose led to a significant suppression of the antioxidant transcription factor Nuclear factor E2 related factor-2 (Nrf2) mediated intracellular pathway, which is demonstrated by the decrease in Nrf2 and increase in Smad3 expression (Fig. 6). Interestingly, we did not detect significant changes in TGF-β1 expression in our studies (see Supplementary Fig. S4).

## Discussion

This study aimed to test acute and chronic effects of an endogenous ligand of cannabinoid receptors AEA on blood pressure and kidney injury in vivo in conscious Dahl SS rats. AEA had no effects on blood pressure in Dahl SS rats on a NS diet. However, prolonged exposure to a high dose of AEA led to the aggravation of SS hypertension and altered kidney functions. AEA could potentially regulate sodium transport at the loop of Henle, where it blocks the Na^+^/H^+^ exchanger and the NKCC cotransporter^6^. Renal intramedullary infusion of AEA induced urinary sodium excretion through metabolism of AEA to prostamides by COX-2^14,40^. Therefore, in our study, a high dose of AEA induced elevated fractional excretion of calcium only.

We identified a significant progression of hypertension in a group treated with a high dose of AEA. The effects on blood pressure were accompanied by a prominent increase in renal interstitial fibrosis and glomerular damage.CB1 receptors are known to play a pivotal role in developing fibrosis in many organs, including liver and kidneys^18,41,42^. Lecru et al. identified that genetic ablation of Cnr1 and CB1 pharmacological blockade dramatically decreased the development of fibrosis in mice during unilateral ureteral obstruction, suggesting the role of CB1 in the development of renal fibrosis^41,43,44^. Thus, treatment with the CB1 receptor antagonist was associated with a significant reduction of kidney injury markers (TIMP-1, KIM-1), in agreement with the substantial decrease of fibrosis in the liver^41,43^. In experimental type 1 diabetes, the CB1 receptor was shown to be overexpressed in podocytes. CB1 receptor blockade ameliorated albuminuria most likely by prevention of nephrin, podocin, and ZO-1 loss^45^. Protein expression of CB1 was significantly enhanced in kidney biopsies of patients with IgA nephropathy, diabetes, and acute interstitial nephritis^44^. Several groups elucidated the role of CB2 receptors in the pathogenesis of renal fibrosis^46–50^. Contrary to CB1 receptors, CB2 deletion exacerbated tissue damage by increasing inflammatory, oxidative, and fibrotic processes not only in the kidney but also liver and skin^51–53^. CB2 inhibition or pharmacological blockade promotes cardiac, liver, and skin fibrosis, while CB2 agonists decrease fibrogenesis^52,54,55^. CB2 agonist SMM-295 was shown to promote renal vasodilatation by activating vascular and nonvascular CB2 receptors and could potentially be used to treat renal injuries that impact renal blood flow dynamics^56^ and AKI^57.^ Different studies showed that CB2 receptor activation exerted anti-inflammatory activity and reduced oxidative/nitrative tissue injury both in liver and kidneys^58–61^. Specific CB2 agonist AM1241 reduced albuminuria by preventing loss of podocyte proteins^48,56,57^. CB2 antagonist SR144528 alone aggravated the development of kidney fibrosis while CB2 agonist JWH133 was shown to diminish the development of renal fibrosis during unilateral ureteral obstruction^44^. Barutta et al. revealed that deletion of CB2 receptors during diabetic nephropathy worsened kidney function in a CB2 knockout mice model^49^. Absence of CB2 receptors on resident glomerular cells had a major role in the progression of diabetic nephropathy, most likely due to enhanced CB1 signaling^49^. A separate report by Zhou et al*.* proposed that the β-arrestin 1-mediated CB2/β-catenin pathway is involved in the development of renal fibrotic injuries^47^. CB2 expression was closely correlated with the progression of kidney fibrosis and was accompanied by the activation of β-catenin. However, this particular signaling pathway needs additional investigation. In the current study we tested several antibodies to detect CB1 and CB2 protein expression (data not shown), but all of them lacked specificity and were therefore unreliable.

AEA-induced blood pressure elevation could be developed through CB1-mediated pressor response. A CB1 receptor inverse agonist was shown to slightly increase and prolong the pressor effect of AEA, suggesting that this pressor effect is partially masked by peripheral CB1 receptors, leading to an inhibition of noradrenaline release^62^. AEA content increased in response to blood pressure elevation in a mid-brain region in Sprague Dawley rats and significantly prolonged the baroreflex-mediated inhibition of the renal sympathetic nerve, leading to prolonged CB1 receptor activation^63^. Some conclusions indicated that ventral medial prefrontal cortex CB1 and TRPV1 receptors could facilitate the cardiac baroreflex activity by either stimulating or blocking NMDA activation and NO synthesis^64^. Dahl SS rats, used in the current research, are known to be associated with baroreflex control system dysfunction^65^. Similar to the spontaneously hypertensive rats, afferent baroreceptor nerve fiber in the SS rat is characterized by elevated pressure thresholds and reduced pressure sensitivity^66^. The alternative pathways of AEA-mediated blood pressure response, including the interplay between the renin-angiotensin system and the ECS, suggest that AEA regulates vascular contraction caused by Ang II. Renin–angiotensin system-ECS interactions may contribute to the enhanced vascular reactivity in early stages of hypertensive pregnancy^67^. AEA reduced Ang II-mediated contraction in a CB1-independent manner^67^. AEA caused a brief rise in blood pressure through renal and mesenteric vasoconstriction after administration to conscious Sprague–Dawley rats, and the CB1 receptor antagonist did not affect any of those cardiovascular effects^68^. Alternatively, eicosanoids can also influence vascular and renal mechanisms of blood pressure regulation^69^. AEA is converted into AA by the fatty acid amide hydrolase (FAAH), which is then metabolized by P450s to vasoactive eicosanoids^26,44,70,71^. This pathway may also affect AEA function. Indeed, inhibition of FAAH increased the bioavailability of AEA and dramatically reduced blood pressure in hypertensive rats^30^.

Other likely AEA target candidates such as GPR55, MAGL, ABHD6, and TRPV1 have only recently been recognized to explain the effects, or lack, thereof in this study. It may be possible that TRPV1, GPR18, or GPR55 could be activated to promote changes in the systemic and regional vasculature either alone or in concert with the non-CB1 receptors^72–75^. It was shown that in dissociated nodose ganglion neurons, AEA produced robust calcium influx through TRPV1 channels^75^. Methanandamide, a stable analog of AEA, potentiated the mechanosensitivity of mucosal bladder afferents, and the effect of mAEA switched from excitatory to inhibitory in the presence of TRPV1 antagonist^76^. CB1 receptors exhibited substantial neuroprotection through the diminished intracellular calcium flux. WIN 55,212–2, another CB1 receptor ligand, inhibited the NMDA-induced increase in intracellular calcium concentration^77^. However, in our studies, the application of AEA did not produce calcium flux in podocytes of freshly isolated glomeruli from SS rats on a NS diet (Supplementary Fig. S5).

A variety of signal transduction pathways are implicated in AEA-induced kidney injury, including the β-arrestin signaling pathway and cannabinoid receptors. β-arrestin 1 is responsible for signal transduction in the CB1 and CB2 receptor intracellular pathway^20,21,78^. In our study, we measured β-arrestin 1 expression in the AEA-treated group. However, neither β-arrestin 1 nor its phosphorylated form were altered during the treatment. Li et al*.* demonstrated that CB2 antagonist AM630 increased collagen deposition and expression levels of TGF-β1 and Smad3 during skin wound repair in the mouse^79^. In primary culture of adult human fibroblasts or in myofibroblasts stimulation with TGF-β increases CB1 receptor expression^44,49,80^. Nrf2 has also been shown to protect against renal fibrosis by inhibiting TGF-β signaling^81^. The stimulation of CB2 signaling by AM1241 diminished the development of myocardial fibrosis during the post-myocardial infarction phase via enhancing the translocation of the fibrogenesis-associated transcription factor Nrf2 to the nucleus and blocking the TGF-β1/Smad3 pathway^82^.

Here we provide evidence of the negative impact of chronic cannabinoid use in the development of kidney damage, especially in hypertensive and CKD patients. Overall, our study shows that prolonged treatment with a high dose of AEA contributes to the aggravation of SS hypertension and a consequent kidney injury, which in turn activates Nrf2-modulated TGF-β1/Smad3 pro-fibrotic signaling^79,82^. We further hypothesize that the Smad complexes translocate to the nucleus and mediate transcription of the genes that contribute to fibrosis, as previously reported^81^. Given that CB receptors are members of the GPCR family, they could also modulate the pro-fibrotic signaling pathway through an alternative TGF-β1 independent pattern^27,29,70,83,84^. Additional research on P450s that directly affect AEA to produce HETE and EET ethanolamides should be conducted in conditions of SS hypertension. It would be of interest to apply the knowledge on the AEA metabolites toward our understanding of the deleterious mechanism of CB receptor activation in the development of fibrosis, because many P450 isoforms have been found to be down-regulated during chronic liver disease^85,86^.

## Material and methods

### Animals and protocols

Eight-week-old male Dahl Salt Sensitive rats (SS; SS/JrHsdMcwi) were provided a normal (0.4% NaCl, # D113755, Dyets Inc.; NS) or a high salt (8% NaCl, # D100078, Dyets Inc.; HS) diets, and water ad libitum. For the surgical procedures, rats were anesthetized with inhalation of 2.5% isoflurane in 0.5 l/min O_2_/N_2_ flow. Catheters (#RPT080 Braintree, MA) were implanted in the femoral artery and vein, tunneled subcutaneously, and exteriorized at the back of the neck in a lightweight tethering spring. Following recovery from anesthesia, all rats were placed into individual cages. The catheters were connected to a pressure transducer (#041516504A, Argon Medical Devices, TX) via swivels (#375/D/22, Instech, PA) for arterial blood pressure acquisition and daily *i.v.* bolus drug infusion. This approach allows the collection of blood samples in conscious animals during the study. The rats were allowed to recover for at least 3 days following surgery. Anandamide (AEA, Cayman chemical, #90,050) was used at a dose 0.05 mg/kg (low dose) or 3 mg/kg (high dose). Rats were treated with AEA or a corresponding vehicle every morning for the whole duration of the experiment. To induce hypertension rats were switched from a NS to a HS diet for 14 days. Urine and blood samples were collected on day 7 and 14 on HS. Treatment with AEA was initiated from day 0 (NS, no treatment). The animal use and welfare adhered to the National Institutes of Health (NIH) Guide for the Care and Use of Laboratory Animals following protocols reviewed and approved by the Medical College of Wisconsin Institutional Animal Care and Use Committee. All experiments were carried out in accordance with relevant guidelines and regulations and in compliance with the ARRIVE guidelines^87^.

### Kidney isolation and glomerular analysis

By the end of the study, kidney tissue was harvested and weighted. Rats were anesthetized, and their kidneys were flushed with phosphate buffered saline (PBS) via aortic catheterization (3 ml/min/kidney until blanched). For each rat, one kidney was used for glomeruli isolation, and the other kidney coronal sections were used for Western blot. Rat kidneys were formalin fixed, paraffin embedded, sectioned, and mounted on slides. Slides were stained with Masson’s trichrome stain^88^. A glomerular injury score was blindly assessed using a 0–4 scale, where 0 = no damage. A cumulative distribution plot was generated in Origin Pro 9.0 (OriginLab, Northampton, MA) using obtained glomerular injury scores, and the probability for a corresponding score interval was calculated. Medullary protein cast analysis was performed using a color thresholding method involving Metamorph software (Molecular Devices, Sunnyvale, CA). Cortical fibrosis was blindly assessed using color deconvolution in the Fiji image application (ImageJ 1. 47v, NIH).

### Electrolyte measurements and albuminuria assay

For urine collection, rats were placed in metabolic cages (no. 40615, Laboratory Products) for a 24-h urine collection. These urine samples were used to determine electrolytes, microalbumin, and creatinine levels. Urinary ion excretion and fractional excretion were calculated as described previously^89^. Whole blood and urine electrolytes and creatinine were measured with a blood gas and electrolyte analyzer (ABL system 800 Flex, Radiometer, Copenhagen, Denmark). Kidney function was determined by measuring albuminuria using a fluorescent assay (Albumin Blue 580 dye, Molecular Probes, Eugene, OR) read by a fluorescent plate reader (FL600, Bio-Tek, Winooski, VT).

### Calcium imaging freshly isolated glomeruli from rats

The laser scanning confocal microscope system Nikon A1-R was used to detect [Ca^2+^]_i_ transients. Samples were imaged using 20 × and 60 × objective lenses: Plan Apo 20x/NA 0.75 and 60x/NA 1.4 Oil. Open-source software ImageJ was used for analysis. Changes in [Ca^2+^]_i_ concentration in isolated glomeruli were estimated by fluorescent dyes: Fluo 4 (ex. 488 em. 520/20 nm; #20,190,588, Invitrogen, OR) and Fura Red (ex. 488 em. > 600 nm; #21,046, AAT Bioquest, Inc, CA).

### Western blot analyses

Changes in protein expression in renal cortex homogenates or urine samples were assessed using primary antibodies (nephrin, 1:1000, #ab58968, Abcam; β arrestin 1 1:1000, #A0998, Abclonal; phospho-β arrestin 1, 1:1000, # 2416, Cell Signaling; p44/42 MAPK (Erk1/2), 1:1000, #9102, Cell Signaling; phospho-p44/42 MAPK (Erk1/2) (Thr202/Tyr204), 1:1000, #9101 Cell Signaling; JNK1/JNK2/JNK3, 1:1000, #PA5-36,548, ThermoFisher; phospho-SAPK/JNK (Thr183/Tyr185) (81E11), 1:1000, #4668 Cell Signaling; Nrf2 1:500, #A1244, Abclonal; TGF-β1 1:500 # A18692; Smad3, 1:500, #A19115, Abclonal. The membranes were blocked with 2% BSA in Tris-buffered saline (TBS) and 0.01% Tween 20 overnight at room temperature and then incubated with primary antibody overnight at room temperature. Secondary antibody (1:10,000) was diluted in 2% BSA in TBS and 0.01% Tween 20, and membranes were incubated at room temperature for 1 h. Protein loading was assessed by immunoblotting using rabbit anti-actin, 1:10,000, Cell Signaling; mouse anti-GAPDH (0411), 1:5000, #sc-47724, SantaCruz; mouse anti-tubulin, 1:10,000, #AC030, Abclonal antibodies.

### Software

Fiji (ImageJ 1.47v)National Institute of Health, USAhttps://imagej.nih.gov/ij/.

SigmaPlot 12.5Systat Software, Inchttps://systatsoftware.com/.

OriginPro 9.0 OriginLab Corporationhttps://www.originlab.com/.

GraphPad Prism 7GraphPad Softwarehttps://www.graphpad.com/.

Metamorph softwareMolecular Devices, LLChttps://www.metamorphsoftware.com/.

### Statistical analysis

Data are presented as means ± SE. In the scatter dot plot, the error bars are set to SEM. Statistical analysis consisted of one-way ANOVA or other if indicated (SigmaPlot 12.5 and GraphPad Prism 7). Differences were considered statistically significant at *p*⩽0.05.

## Supplementary Information

Supplementary Information.

## References

[1] Maccarrone M (2015). Endocannabinoid signaling at the periphery: 50 years after THC. Trends Pharmacol. Sci..

[2] Luchicchi A, Pistis M (2012). Anandamide and 2-arachidonoylglycerol: pharmacological properties, functional features, and emerging specificities of the two major endocannabinoids. Mol. Neurobiol..

[3] Mechoulam R (1995). Identification of an endogenous 2-monoglyceride, present in canine gut, that binds to cannabinoid receptors. Biochem. Pharmacol..

[4] Hanus L (2001). 2-arachidonyl glyceryl ether, an endogenous agonist of the cannabinoid CB1 receptor. Proc. Natl. Acad. Sci. USA.

[5] Lim JC, Lim SK, Han HJ, Park SH (2010). Cannabinoid receptor 1 mediates palmitic acid-induced apoptosis via endoplasmic reticulum stress in human renal proximal tubular cells. J. Cell Physiol..

[6] Silva GB, Atchison DK, Juncos LI, Garcia NH (2013). Anandamide inhibits transport-related oxygen consumption in the loop of Henle by activating CB1 receptors. Am. J. Physiol. Renal. Physiol..

[7] Larrinaga G (2010). Expression of cannabinoid receptors in human kidney. Histol. Histopathol..

[8] Lim JC (2011). Cannabinoid receptor 1 mediates high glucose-induced apoptosis via endoplasmic reticulum stress in primary cultured rat mesangial cells. Am. J. Physiol. Renal. Physiol..

[9] Lim SK, Park SH (2012). The high glucose-induced stimulation of B1R and B2R expression via CB(1)R activation is involved in rat podocyte apoptosis. Life Sci..

[10] Deutsch DG (1997). Production and physiological actions of anandamide in the vasculature of the rat kidney. J. Clin. Investig..

[11] Jenkin KA, McAinch AJ, Grinfeld E, Hryciw DH (2010). Role for cannabinoid receptors in human proximal tubular hypertrophy. Cell Physiol Biochem.

[12] Jenkin KA (2016). Renal effects of chronic pharmacological manipulation of CB2 receptors in rats with diet-induced obesity. Br. J. Pharmacol..

[13] Jenkin KA (2013). Cannabinoid receptor 2 expression in human proximal tubule cells is regulated by albumin independent of ERK1/2 signaling. Cell Physiol. Biochem..

[14] Ritter JK (2012). Production and actions of the anandamide metabolite prostamide E2 in the renal medulla. J. Pharmacol. Exp. Ther..

[15] Li GB (2016). Protective Action of Anandamide and Its COX-2 Metabolite against L-Homocysteine-Induced NLRP3 Inflammasome Activation and Injury in Podocytes. J. Pharmacol. Exp. Ther..

[16] Ritter JK, Li G, Xia M, Boini K (2016). Anandamide and its metabolites: what are their roles in the kidney?. Front Biosci. (Schol Ed.).

[17] Barutta F, Mastrocola R, Bellini S, Bruno G, Gruden G (2018). Cannabinoid receptors in diabetic kidney disease. Curr. Diab. Rep..

[18] Tam J (2016). The emerging role of the endocannabinoid system in the pathogenesis and treatment of kidney diseases. J. Basic Clin. Physiol. Pharmacol..

[19] Sampaio LS (2015). The endocannabinoid system in renal cells: regulation of Na+ transport by CB1 receptors through distinct cell signalling pathways. Brit. J. Pharmacol..

[20] Nogueras-Ortiz C (2017). Retromer stops beta-arrestin 1-mediated signaling from internalized cannabinoid 2 receptors. Mol. Biol. Cell.

[21] Ahn KH, Mahmoud MM, Shim J-Y, Kendall DA (2013). Distinct roles of beta-arrestin 1 and beta-arrestin 2 in ORG27569-induced biased signaling and internalization of the cannabinoid receptor 1 (CB1). J. Biol. Chem..

[22] Munro S, Thomas KL, Abu-Shaar M (1993). Molecular characterization of a peripheral receptor for cannabinoids. Nature.

[23] Snider NT, Kornilov AM, Kent UM, Hollenberg PF (2007). Anandamide metabolism by human liver and kidney microsomal cytochrome p450 enzymes to form hydroxyeicosatetraenoic and epoxyeicosatrienoic acid ethanolamides. J. Pharmacol. Exp. Ther..

[24] Spector AA, Norris AW (2007). Action of epoxyeicosatrienoic acids on cellular function. Am. J. Physiol. Cell Physiol..

[25] Muralikrishna Adibhatla, R. & Hatcher, J.F. Phospholipase A2, reactive oxygen species, and lipid peroxidation in cerebral ischemia. *Free Radic Biol Med* **40**, 376–387 (2006). 10.1016/j.freeradbiomed.2005.08.044 16443152

[26] Cravatt BF (2004). Functional disassociation of the central and peripheral fatty acid amide signaling systems. Proc. Natl. Acad. Sci. USA.

[27] Zelasko S, Arnold WR, Das A (2015). Endocannabinoid metabolism by cytochrome P450 monooxygenases. Prostaglandins Other Lipid Mediat..

[28] Van Der Stelt M (2000). Formation of a new class of oxylipins from N-acyl(ethanol)amines by the lipoxygenase pathway. Eur. J. Biochem..

[29] Jin M, Kumar A, Kumar S (2012). Ethanol-mediated regulation of cytochrome P450 2A6 expression in monocytes: role of oxidative stress-mediated PKC/MEK/Nrf2 pathway. PLoS ONE.

[30] Batkai S (2004). Endocannabinoids acting at cannabinoid-1 receptors regulate cardiovascular function in hypertension. Circulation.

[31] Jarai Z (1999). Cannabinoid-induced mesenteric vasodilation through an endothelial site distinct from CB1 or CB2 receptors. Proc. Natl. Acad. Sci. USA.

[32] Kunos G (2000). Endocannabinoids as cardiovascular modulators. Chem. Phys. Lipids.

[33] Pacher P, Batkai S, Kunos G (2005). Blood pressure regulation by endocannabinoids and their receptors. Neuropharmacology.

[34] Kanakis C, Pouget JM, Rosen KM (1976). The effects of delta-9-tetrahydrocannabinol (cannabis) on cardiac performance with and without beta blockade. Circulation.

[35] Benowitz NL, Jones RT (1975). Cardiovascular effects of prolonged delta-9-tetrahydrocannabinol ingestion. Clin. Pharmacol. Ther..

[36] Varga K, Lake KD, Huangfu D, Guyenet PG, Kunos G (1996). Mechanism of the hypotensive action of anandamide in anesthetized rats. Hypertension.

[37] Garcia N, Jarai Z, Mirshahi F, Kunos G, Sanyal AJ (2001). Systemic and portal hemodynamic effects of anandamide. Am. J. Physiol. Gastrointest. Liver Physiol..

[38] Ho WS, Gardiner SM (2009). Acute hypertension reveals depressor and vasodilator effects of cannabinoids in conscious rats. Br. J. Pharmacol..

[39] Koura Y (2004). Anandamide decreases glomerular filtration rate through predominant vasodilation of efferent arterioles in rat kidneys. J Am Soc Nephrol.

[40] Ahmad A (2017). Stimulation of diuresis and natriuresis by renomedullary infusion of a dual inhibitor of fatty acid amide hydrolase and monoacylglycerol lipase. Am. J. Physiol. Renal Physiol..

[41] Udi S (2017). Proximal Tubular Cannabinoid-1 Receptor Regulates Obesity-Induced CKD. J. Am. Soc. Nephrol..

[42] Wu HM, Yang YM, Kim SG (2011). Rimonabant, a Cannabinoid Receptor Type 1 Inverse Agonist, Inhibits Hepatocyte Lipogenesis by Activating Liver Kinase B1 and AMP-Activated Protein Kinase Axis Downstream of G alpha(i/o) Inhibition. Mol. Pharmacol..

[43] Giannone FA (2012). Reversal of liver fibrosis by the antagonism of endocannabinoid CB1 receptor in a rat model of CCl(4)-induced advanced cirrhosis. Lab Invest.

[44] Lecru L (2015). Cannabinoid receptor 1 is a major mediator of renal fibrosis. Kidney Int..

[45] Barutta F (2010). Cannabinoid receptor 1 blockade ameliorates albuminuria in experimental diabetic nephropathy. Diabetes.

[46] Zhou L (2018). Targeted inhibition of the type 2 cannabinoid receptor is a novel approach to reduce renal fibrosis. Kidney Int.

[47] Zhou S (2021). Cannabinoid receptor type 2 promotes kidney fibrosis through orchestrating beta-catenin signaling. Kidney Int..

[48] Barutta F (2011). Protective role of cannabinoid receptor type 2 in a mouse model of diabetic nephropathy. Diabetes.

[49] Barutta F (2014). Deficiency of cannabinoid receptor of type 2 worsens renal functional and structural abnormalities in streptozotocin-induced diabetic mice. Kidney Int..

[50] Barutta, F., Mastrocola, R., Bellini, S., Bruno, G. & Gruden, G. Cannabinoid Receptors in Diabetic Kidney Disease. *Curr. Diabetes Rep.* **18**(2018). 10.1007/s11892-018-0975-7 29399721

[51] Akhmetshina A (2009). The cannabinoid receptor CB2 exerts antifibrotic effects in experimental dermal fibrosis. Arthritis Rheum.

[52] Julien B (2005). Antifibrogenic role of the cannabinoid receptor CB2 in the liver. Gastroenterology.

[53] Munoz-Luque J (2008). Regression of fibrosis after chronic stimulation of cannabinoid CB2 receptor in cirrhotic rats. J. Pharmacol. Exp. Ther..

[54] Defer N (2009). The cannabinoid receptor type 2 promotes cardiac myocyte and fibroblast survival and protects against ischemia/reperfusion-induced cardiomyopathy. FASEB J.

[55] Servettaz A (2010). Targeting the cannabinoid pathway limits the development of fibrosis and autoimmunity in a mouse model of systemic sclerosis. Am. J. Pathol..

[56] Pressly JD (2019). Activation of the cannabinoid receptor 2 increases renal perfusion. Physiol. Genomics.

[57] Pressly JD (2018). Selective Cannabinoid 2 receptor stimulation reduces tubular epithelial cell damage after renal ischemia-reperfusion injury. J. Pharmacol. Exp. Ther..

[58] Trojnar E (2020). Cannabinoid-2 receptor activation ameliorates hepatorenal syndrome. Free Radic. Biol. Med..

[59] Mukhopadhyay P (2010). Cannabinoid-2 receptor limits inflammation, oxidative/nitrosative stress, and cell death in nephropathy. Free Radic. Biol. Med..

[60] Nettekoven M (2016). Novel triazolopyrimidine-derived cannabinoid receptor 2 agonists as potential treatment for inflammatory kidney diseases. ChemMedChem.

[61] Mukhopadhyay P (2016). The novel, orally available and peripherally restricted selective cannabinoid CB2 receptor agonist LEI-101 prevents cisplatin-induced nephrotoxicity. Br. J. Pharmacol..

[62] Lake KD, Martin BR, Kunos G, Varga K (1997). Cardiovascular effects of anandamide in anesthetized and conscious normotensive and hypertensive rats. Hypertension.

[63] Brozoski DT, Dean C, Hopp FA, Seagard JL (2005). Uptake blockade of endocannabinoids in the NTS modulates baroreflex-evoked sympathoinhibition. Brain Res..

[64] Lagatta DC, Kuntze LB, Ferreira-Junior NC, Resstel LBM (2018). Medial prefrontal cortex TRPV1 and CB1 receptors modulate cardiac baroreflex activity by regulating the NMDA receptor/nitric oxide pathway. Pflugers Arch..

[65] Bugenhagen SM, Cowley AW, Beard DA (2010). Identifying physiological origins of baroreflex dysfunction in salt-sensitive hypertension in the Dahl SS rat. Physiol. Genom..

[66] Andresen MC, Rudis SK, Bee DE (1989). Aberrant baroreceptor mechanotransduction in adult Dahl rats on low-salt diet. Am. J. Physiol..

[67] Pulgar VM (2014). Increased Angiotensin II contraction of the uterine artery at early gestation in a transgenic model of hypertensive pregnancy is reduced by inhibition of endocannabinoid hydrolysis. Hypertension.

[68] Gardiner SM, March JE, Kemp PA, Bennett T (2002). Complex regional haemodynamic effects of anandamide in conscious rats. Br. J. Pharmacol..

[69] Nasjletti, A. Arthur C. Corcoran Memorial Lecture. The role of eicosanoids in angiotensin-dependent hypertension. *Hypertension* **31**, 194–200 (1998). 10.1161/01.hyp.31.1.194 9453302

[70] Watanabe H (2003). Anandamide and arachidonic acid use epoxyeicosatrienoic acids to activate TRPV4 channels. Nature.

[71] Imig JD (2006). Eicosanoids and renal vascular function in diseases. Clin. Sci. (Lond).

[72] Akerman S, Kaube H, Goadsby PJ (2004). Anandamide acts as a vasodilator of dural blood vessels in vivo by activating TRPV1 receptors. Brit. J. Pharmacol..

[73] Johns DG (2007). The novel endocannabinoid receptor GPR55 is activated by atypical cannabinoids but does not mediate their vasodilator effects. Brit. J. Pharmacol..

[74] Penumarti A, Abdel-Rahman AA (2014). The novel endocannabinoid receptor GPR18 Is expressed in the rostral ventrolateral medulla and exerts tonic restraining influence on blood pressure. J. Pharmacol. Exp. Ther..

[75] Fenwick AJ (2017). Direct anandamide activation of TRPV1 produces divergent calcium and current responses. Front Mol. Neurosci..

[76] Christie S, Zagorodnyuk V (2021). CB2 cannabinoid receptor agonist selectively inhibits the mechanosensitivity of mucosal afferents in the guinea pig bladder. Am. J. Physiol. Renal. Physiol..

[77] Zhuang SY (2005). Cannabinoids produce neuroprotection by reducing intracellular calcium release from ryanodine-sensitive stores. Neuropharmacology.

[78] Blume LC (2017). Cannabinoid receptor interacting protein 1a competition with beta-Arrestin for CB1 receptor binding sites. Mol. Pharmacol..

[79] Li SS (2016). Cannabinoid CB2 receptors are involved in the regulation of fibrogenesis during skin wound repair in mice. Mol. Med. Rep..

[80] Correia-Sa, I.B.*, et al.* AM251, a cannabinoid receptor 1 antagonist, prevents human fibroblasts differentiation and collagen deposition induced by TGF-beta—an in vitro study. *Eur J Pharmacol* **892**(2021). 10.1016/j.ejphar.2020.173738 33220269

[81] Oh CJ (2012). Dimethylfumarate attenuates renal fibrosis via NF-E2-related factor 2-mediated inhibition of transforming growth factor-beta/Smad signaling. PLoS ONE.

[82] Li X (2016). Activation of cannabinoid receptor type II by AM1241 ameliorates myocardial fibrosis via Nrf2-Mediated Inhibition of TGF-beta1/Smad3 pathway in myocardial infarction mice. Cell Physiol. Biochem..

[83] Wang W (2006). Essential role of Smad3 in angiotensin II-induced vascular fibrosis. Circ. Res..

[84] Arnold WR, Weigle AT, Das A (2018). Cross-talk of cannabinoid and endocannabinoid metabolism is mediated via human cardiac CYP2J2. J. Inorg. Biochem..

[85] Yang LQ (2003). Different alterations of cytochrome P450 3A4 isoform and its gene expression in livers of patients with chronic liver diseases. World J. Gastroenterol..

[86] Horiike N (2005). The quantification of cytochrome P-450 (CYP 3A4) mRNA in the blood of patients with viral liver diseases. Clin Biochem.

[87] Percie du Sert, N.*, et al.* Reporting animal research: Explanation and elaboration for the ARRIVE guidelines 2.0. *PLoS Biol* **18**, e3000411 (2020). 10.1371/journal.pbio.3000411 PMC7360025 32663221

[88] Palygin O (2019). Progression of diabetic kidney disease in T2DN rats. Am. J. Physiol. Renal. Physiol..

[89] Golosova DV, Natochin VV (2017). New approach to calculation of renal cation clearance in rats after V1a receptor stimulation. Bull. Exp. Biol. Med..

