# Supplementary material for: Acute and long-term effects of cannabinoids on hypertension and kidney injury
Source: Sci Rep. 2022 Apr 12;12:6080. doi: 10.1038/s41598-022-09902-6 (PMC9005691; doi:10.1038/s41598-022-09902-6)
Supplement: Supplementary file 1 — Supplementary Information. [file 41598_2022_9902_MOESM1_ESM.docx]

**Online Supplement**

**Acute and Long-Term Effects of Cannabinoids on Hypertension and Kidney Injury**

Daria Golosova^1,*^, Vladislav Levchenko^2^, Olha Kravtsova^2^, Oleg Palygin^1,3^, Alexander Staruschenko^1,2,4,5*^

From the ^1^Department of Physiology, Medical College of Wisconsin, Milwaukee, WI 53226, USA; ^2^Department of Molecular Pharmacology and Physiology, University of South Florida, Tampa, FL 33602, USA; ^3^Division of Nephrology, Department of Medicine, Medical University of South Carolina, Charleston, SC 29425, USA; ^4^Hypertension and Kidney Research Center, University of South Florida, Tampa, FL 33602, USA; ^5^Clement J. Zablocki VA Medical Center, Milwaukee, WI 53295, USA

**^*^To whom correspondence should be addressed**

Alexander Staruschenko, PhD; Department of Molecular Pharmacology and Physiology, University of South Florida, 560 Channelside Dr., Tampa, FL 33602, USA. E-mail: [staruschenko@usf.edu](mailto:staruschenko@usf.edu)

Daria Golosova, MD, PhD; Department of Physiology, Medical College of Wisconsin, 8701 Watertown Plank Road, Milwaukee, WI 53226, USA. Phone: (414) 955-7415; E-mail: [dgolosova@mcw.edu](mailto:staruschenko@mcw.edu)

**Supplementary Table S1**. **Urinary excretion of ions in SS rats with or without chronic treatment with the 0.05 mg/kg of AEA within 14 days on HS.**

| **Urinary ion excretion, mmol per day** | **7DHS+Vehicle** | **7DHS+AEA (0.05 mg/kg)** |
| --- | --- | --- |
| Potassium | 1.02±0.03 | 1.21±0.09 |
| Sodium | 25±1 | 25±2 |
| Calcium | 0.16±0.04 | 0.15±0.02 |
| Chloride | 25±1 | 25±2 |
| **14DHS** | **14DHS+Vehicle** | **14DHS+AEA (0.05 mg/kg)** |
| Potassium | 1.20±0.05 | 1.09±0.11 |
| Sodium | 23.1±0.4 | 18±2 |
| Calcium | 0.15±0.01 | 0.11±0.01 |
| Chloride | 23.0±0.3 | 18±2 |

7DHS, 14DHS – 7 days and 14 days on HS (8% NaCl). Data are presented as means ± SE. n ≥ 5 rats for each group; t-test *P < 0.05. Urine samples were collected for 24 hrs.

**Supplementary Table S2**. **Urinary excretion of ions in SS rats with or without chronic treatment with the 3 mg/kg of AEA within 14 days on HS.**

| **Urinary ion excretion, mmol per day** | **7DHS+Vehicle** | **7DHS+AEA (3 mg/kg)** |
| --- | --- | --- |
| Potassium | 1.10±0.03 | 0.98±0.05 |
| Sodium | 21±1 | 20±1 |
| Calcium | 0.13±0.01 | 0.15±0.02 |
| Chloride | 20±1 | 20±2 |
| **14DHS** | **14DHS+Vehicle** | **14DHS+AEA (3 mg/kg)** |
| Potassium | 1.23±0.04 | 1.05±0.08 |
| Sodium | 22±1 | 20±2 |
| Calcium | 0.16±0.01 | 0.14±0.02 |
| Chloride | 22±1 | 19±2 |

7DHS, 14DHS – 7 days and 14 days on HS (8% NaCl). Data are presented as means ± SE. n ≥ 8 rats for each group; t-test *P < 0.05. Urine samples were collected for 24 hrs.

**
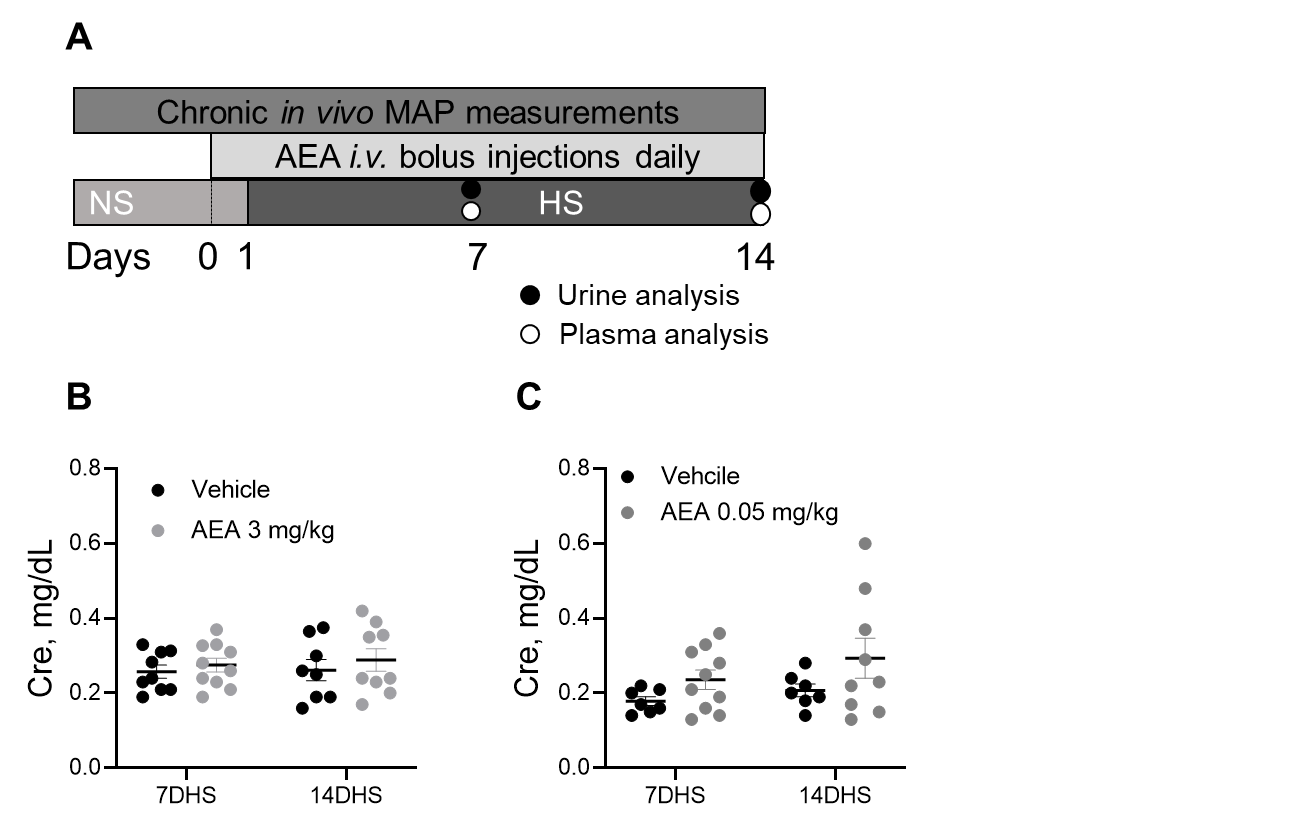
**

**Supplementary Figure S1.** Schematic representation of the experimental protocol. **A.** Chronic treatment with AEA in SS rats following a change in a diet from a normal salt (NS; 0.4 % NaCl) to a high salt (HS; 8% NaCl) and chronically treated with AEA or vehicle. The drug was injected starting day 0 (NS). **B, C.** Plasma creatinine levels in both groups treated with AEA.


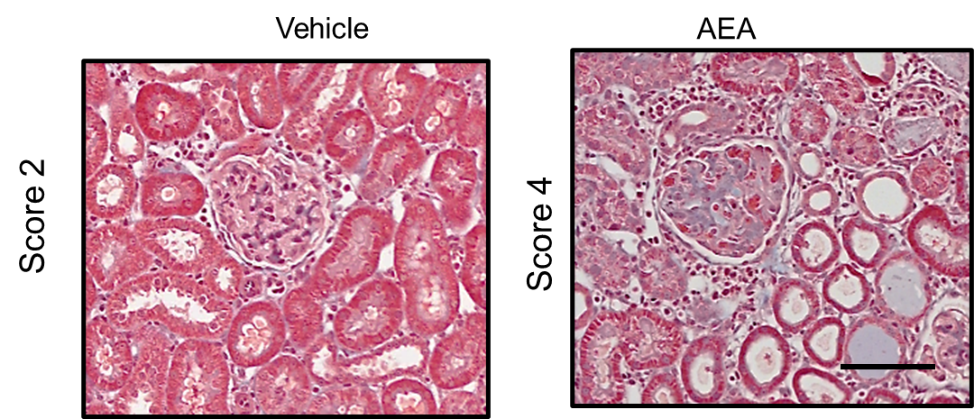


**Supplementary Figure S2.** Representative images of kidney tissue stained with Masson’s trichrome (×40 magnification). Glomeruli damage score in a group treated either with AEA 3mg/kg or a corresponding vehicle. Scale bar is 150 µm.

**
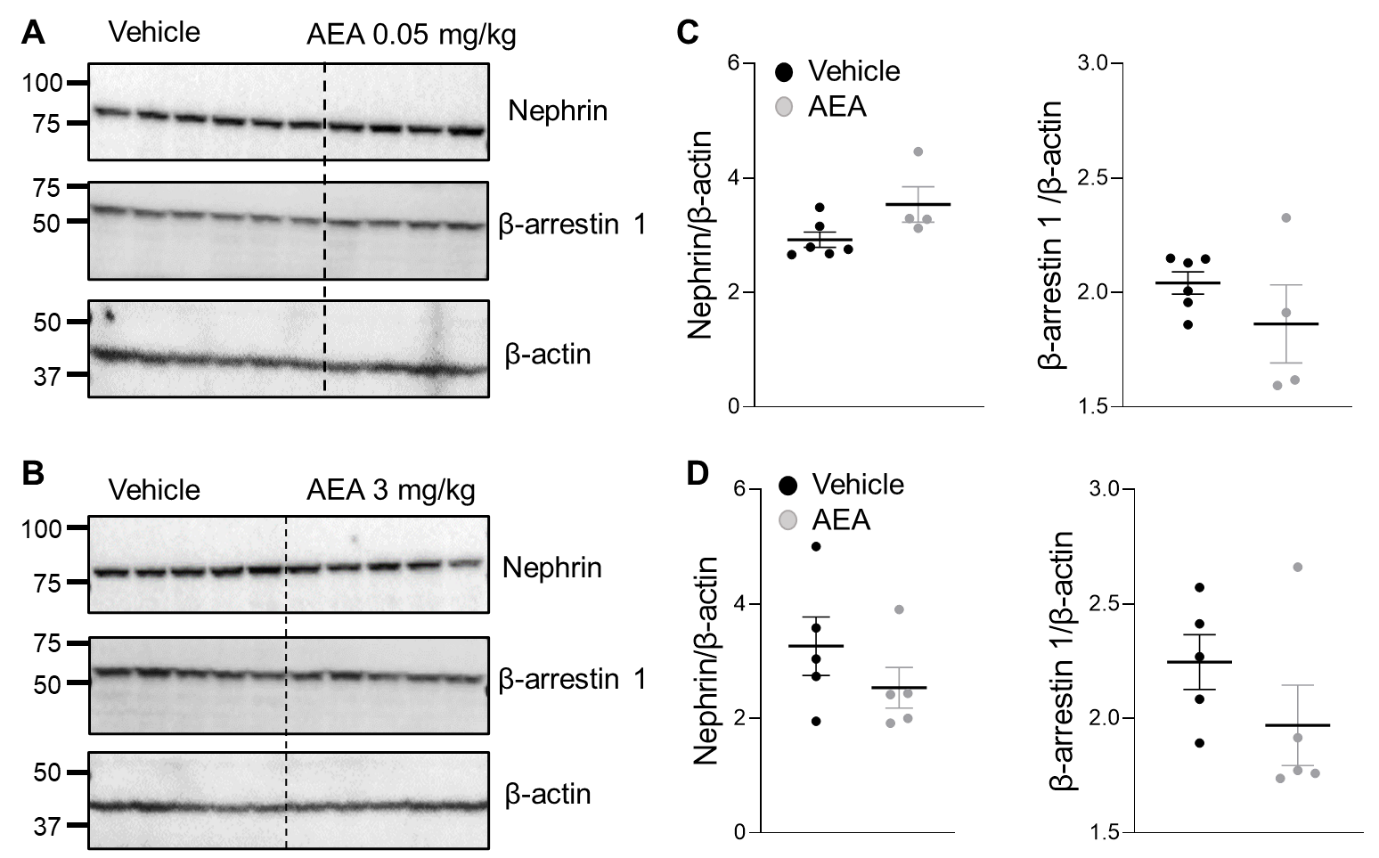
**

**Supplementary Figure S3.** The effect of anandamide on the expression of nephrin and β-arrestin 1 in the kidney. Representative Western blots showing renal expression of nephrin and β-arrestin 1 in the kidney cortex after chronic treatment with AEA at a dose 0.05 mg/kg (**A**) and 3 mg/kg (**B**). The summary quantitative data graphs show changes in nephrin and β-arrestin 1 normalized to β-actin after chronic treatment with AEA at a dose 0.05 mg/kg (**C**) and 3 mg/kg (**D**). Error bars are ± SEM. Full-length blots are available in Supplementary Figure S7.


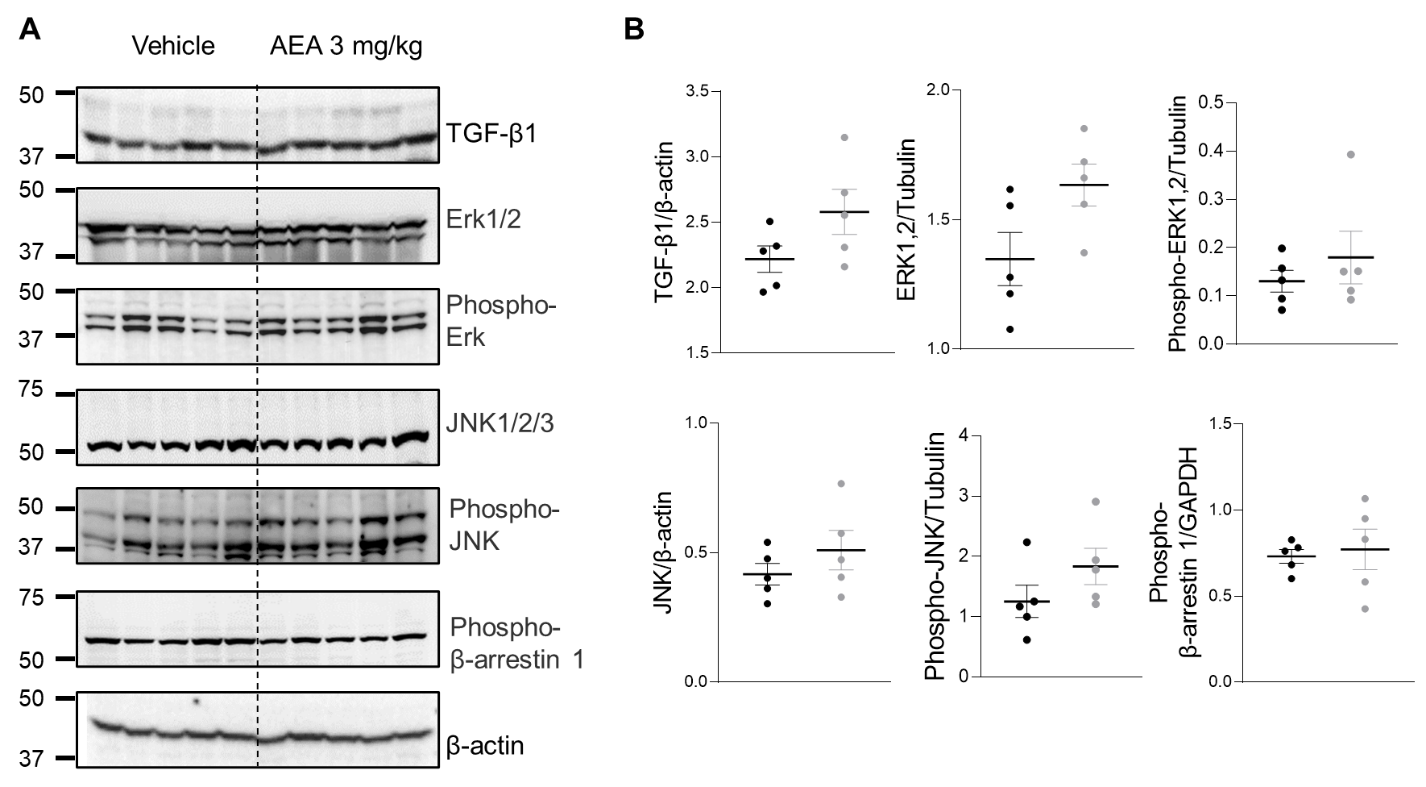


**Supplementary Figure S4. Chronic treatment with AEA at a dose of 3 mg/kg does not lead to any changes in TGF-β1 expression in renal cortex.** (**A**) Western blots showing renal expression of TGF-β1, Erk1/2, JNK1/2/3, phospho-JNK, phospho-β-arrestin 1 after chronic treatment with a high dose of AEA. (**B**) Quantitative data showing renal expression of TGF-β1, Erk1/2, phospho-Erk1/2, JNK1/2/3, phospho-JNK, phospho-β-arrestin 1 normalized to a corresponding loading control in the kidney cortex after chronic treatment with AEA at a dose 3 mg/kg. Uncropped blots are presented in Supplementary Figure S8.


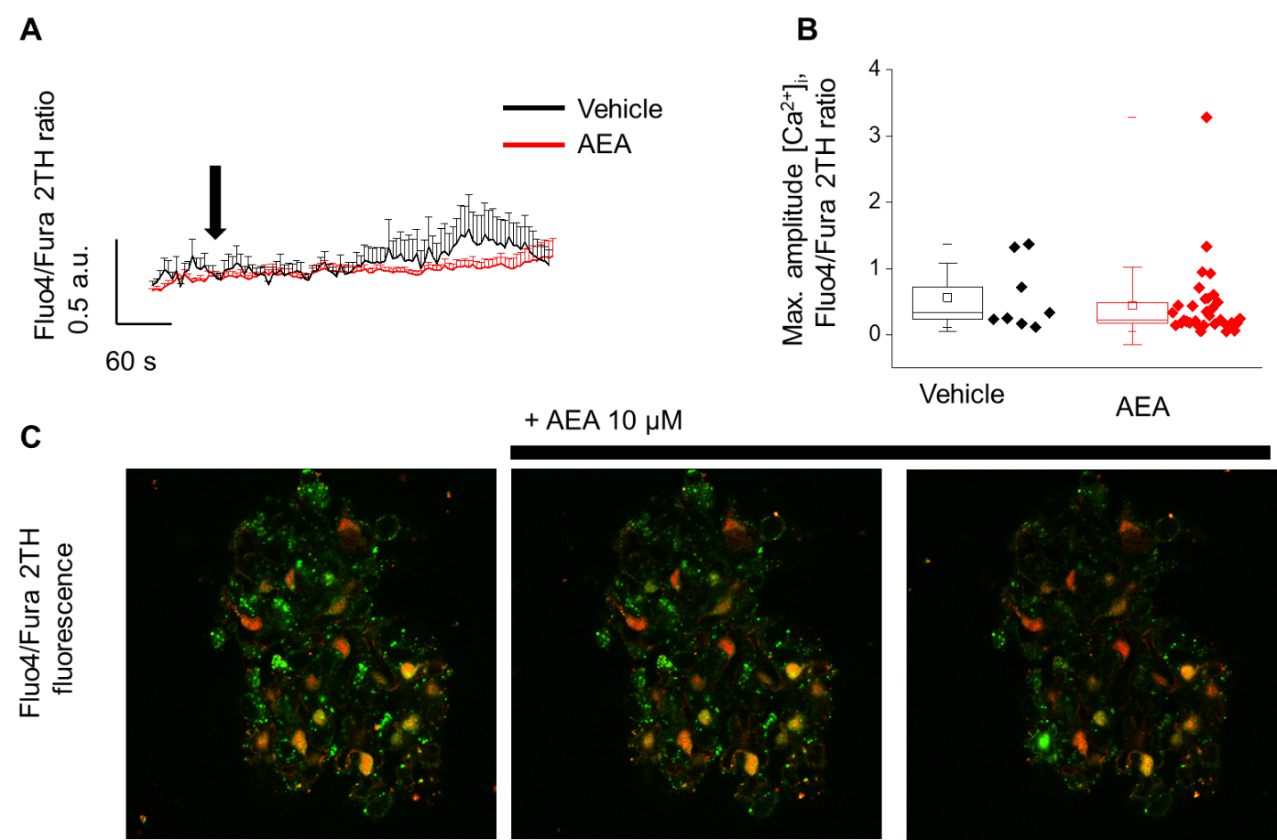


**Supplementary Figure S5.** Acute application of AEA does not change [Ca^2+^]_i_ levels in podocytes from freshly isolated rat glomeruli. **A.** The example of [Ca^2+^]_i_ levels after an application of 10 µM of AEA in podocytes from freshly isolated glomeruli from SS rats. **B**. Summary for the peak fluorescence intensity following an application of AEA. **C.** Representative image of a glomerulus loaded with ratiometric calcium dyes (Fluo4/Fura 2TH; merged) before and after the application of AEA. The ratiometric fluorescence intensity values (Fluo4/Fura 2TH) were calculated by selecting podocytes as regions of interest in Fiji, and baseline correction was performed in OriginPro 9.0.

**
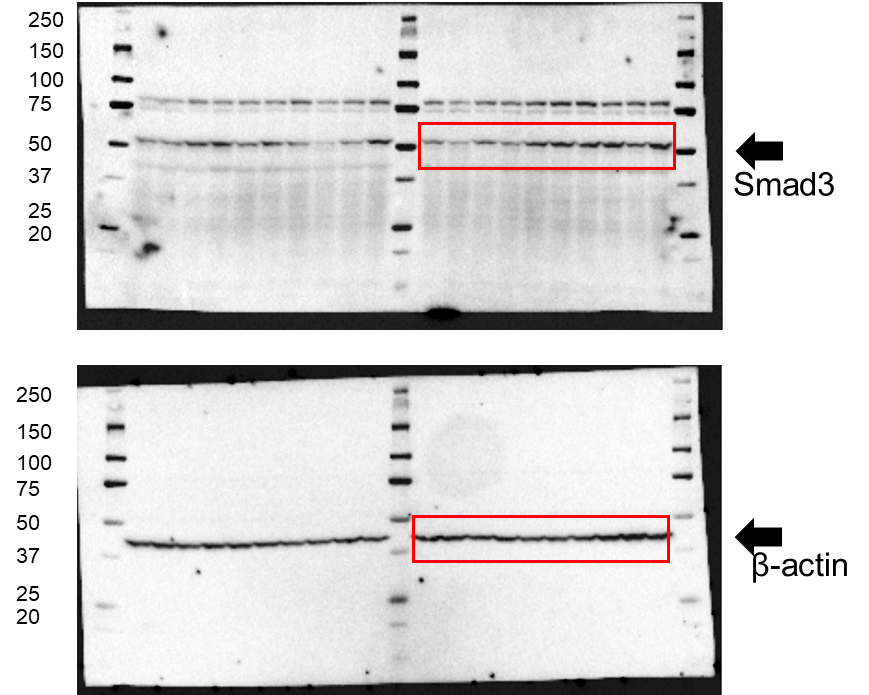
**

**
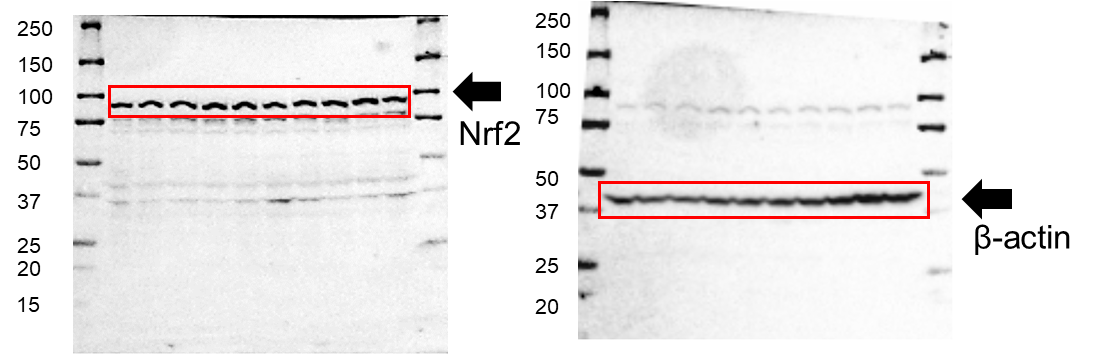
**

**Supplementary Figure S6.** Full-length uncropped blots showing Smad3 and Nrf2 protein expression demonstrated in Figure 6.

**
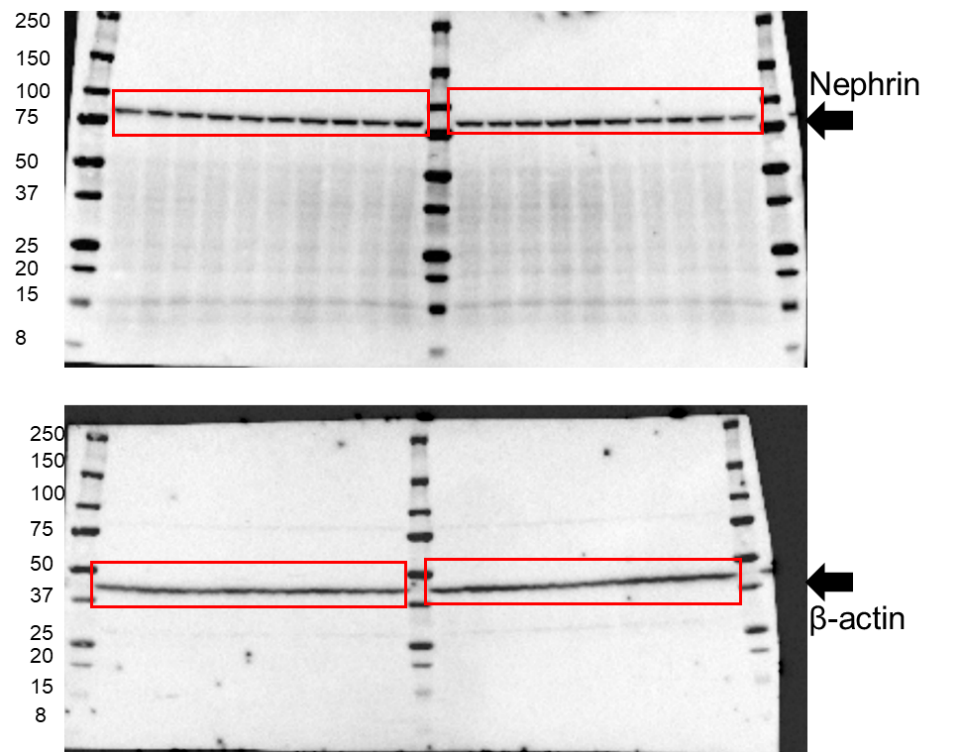
**

**
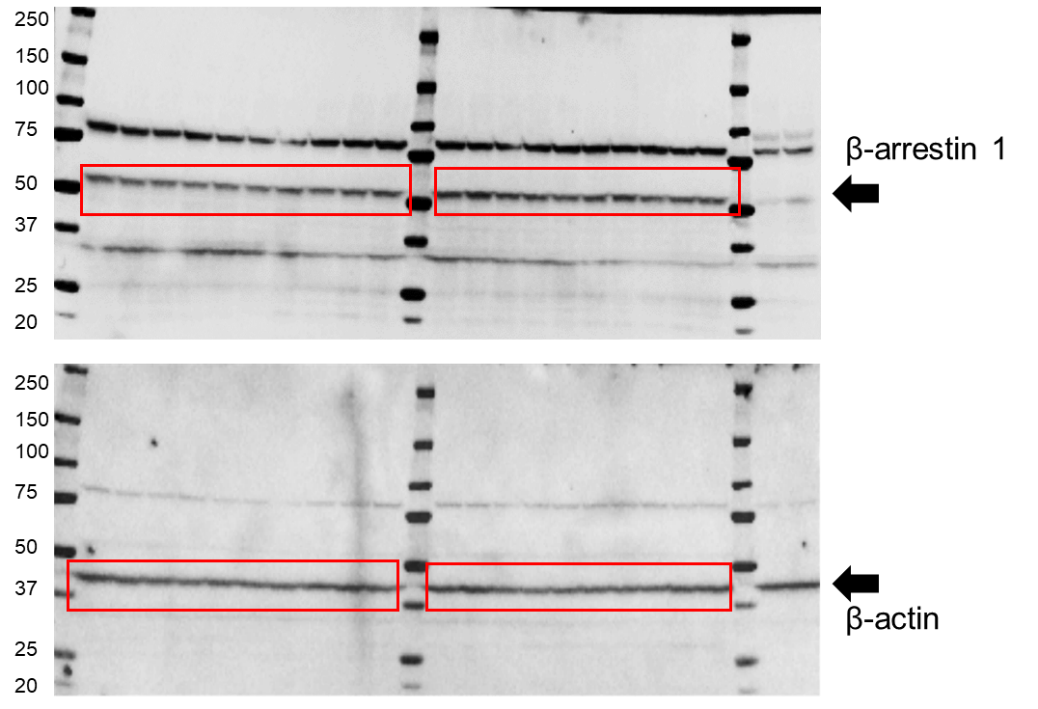
**

**Supplementary Figure S7.** Full-length uncropped blots showing expression of nephrin and β-arrestin 1 in Supplementary Figure S3.


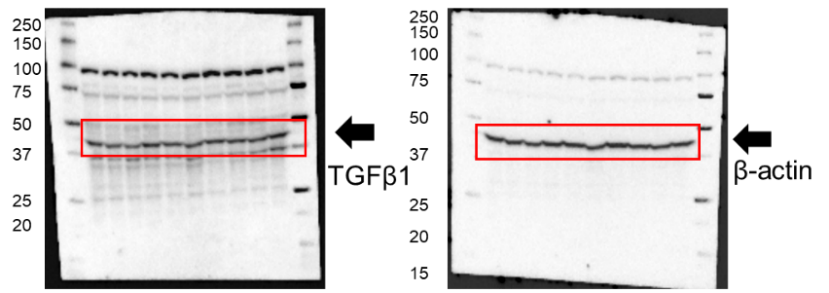

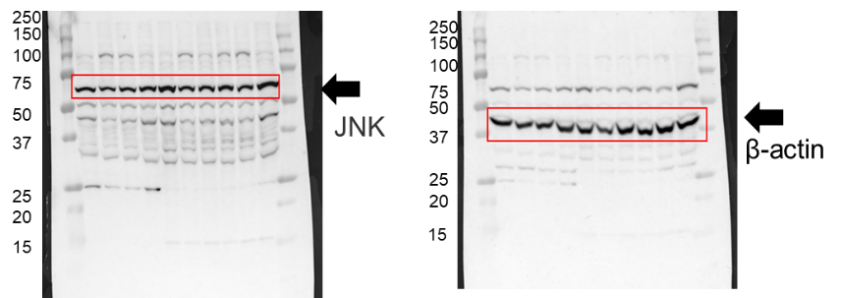

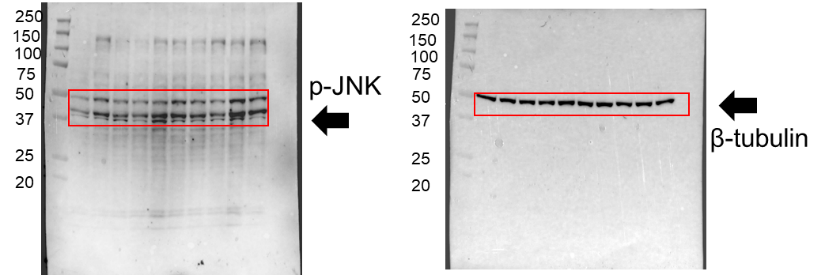

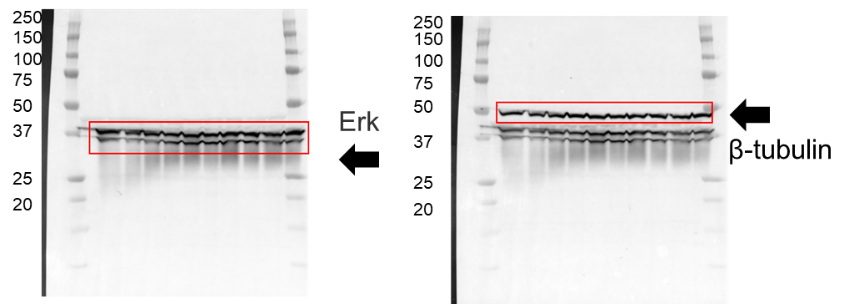

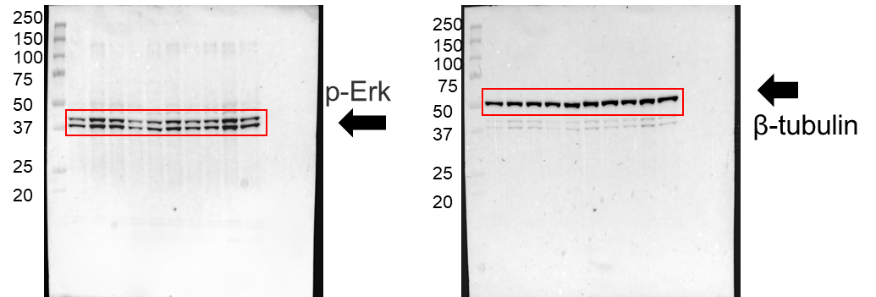

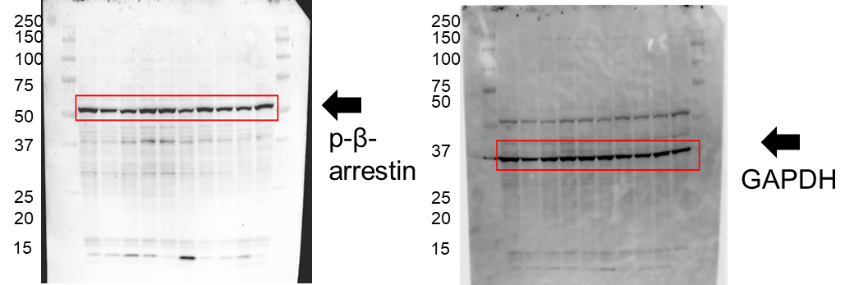


**Supplementary Figure S8.** Full-length uncropped blots showing expression of TGF-β1, TGF-β1, Erk1/2, phospho-Erk1/2, JNK1/2/3, phospho-JNK, phospho-β-arrestin 1 after chronic treatment with a high dose of AEA represented in Supplementary Figure S4
